# Effects of physician visit frequency for Parkinson’s disease treatment on mortality, hospitalization, and costs: a retrospective cohort study

**DOI:** 10.1186/s12877-021-02685-x

**Published:** 2021-12-15

**Authors:** Takako Fujita, Akira Babazono, Sung-a Kim, Aziz Jamal, Yunfei Li

**Affiliations:** 1 Department of Health Sciences, Faculty of Medical Sciences, Kyushu University, 3-1-1 Maidashi, Higashi-ku, Fukuoka, 812-8582 Japan; 2 Department of Healthcare Administration and Management, Graduate School of Medical Sciences, Kyushu University, 3-1-1 Maidashi, Higashi-ku, Fukuoka, 812-8582 Japan; 3 Health Administration Program, Department of International Business and Management, Universiti Teknologi MARA, Selangor Campsus, Shah Alam, Malaysia

**Keywords:** Parkinson’s disease, Physician visit frequency, Mortality, Hospitalization, Costs

## Abstract

**Background:**

The number of patients with Parkinson’s disease among older adults is rapidly increasing. Such patients mostly take medication and require regular physician visits. However, the effect of physician visit frequency for the treatment for Parkinson’s disease has not been evaluated. This study aimed to evaluate the impact of physician visit frequency for Parkinson’s disease treatment on mortality, healthcare days, and healthcare and long-term care costs among older adults.

**Methods:**

This study employed a retrospective cohort design utilizing claims data from the Fukuoka Prefecture Wide-Area Association of Latter-Stage Elderly Healthcare Insurance and Long-Term Care Insurance. Patients aged ≥75 years who were newly diagnosed with Parkinson’s disease in 2014 were included in this study, following the onset of Parkinson’s disease to March 31, 2019. We calculated the restricted mean survival time to evaluate mortality, focusing on the frequency of physician visits for Parkinson’s disease treatment. Inpatient days, outpatient days, and healthcare and long-term care costs per month were calculated using a generalized linear model.

**Results:**

There were 2224 participants, with 46.5% mortality among those with a higher frequency of physician visits and 56.4% among those with a lower frequency of physician visits. A higher frequency of physician visits was associated with a significant increase in survival time (1.57 months at 24 months and 5.00 months at 60 months) after the onset of Parkinson’s disease and a decrease in inpatient days and healthcare costs compared to a lower frequency of physician visits.

**Conclusions:**

A higher frequency of physician visits was significantly associated with longer survival time, fewer inpatient days, and lower healthcare costs. Caregivers should support patients with Parkinson’s disease to visit physicians regularly for their treatment.

## Background

Globally, an estimated 6.1 million people in 2016 suffered from Parkinson’s disease (PD) [1]. According to the 2017 Patient Survey in Japan, 162,000 people, mostly older adults, were estimated to be diagnosed with PD, with approximately 145,000 (89.5%) aged ≥65 years, 105,000 (64.8%) of whom were aged ≥75 years [2].

Despite many studies reporting that PD patients have a reduced life expectancy compared to the general population [3–5], others reported no significant difference [6] and that life expectancy is higher than for other Parkinsonism diseases [7]. One study predicted that the life expectancy of patients with PD will increase in the future [8]. Patients with PD need both healthcare and long-term care to manage the disease’s progression. All individuals in Japan have healthcare insurance, and Japan introduced Long-Term Care Insurance (LTCI) for all individuals aged ≥40 years. PD patients receive LTCI service when they are approved for their long-term care level, which is principally reassessed after 6 months. Long-term care levels are divided into seven categories: requiring help levels 1 and 2, in which long-term care can be avoided if patients receive preventive services, and long-term care levels 1–5. Inclusion in these levels is based on how much individuals need help as examined by the 74 items of the questionnaire [9]. The criteria for inclusion in each of the levels are based on decision trees constructed by the discriminant function analyses [9, 10]. People who require long-term care levels 1 and 2 need some help in their daily lives; those who require long-term care level 3 have difficulty walking by themselves and need complete help in their daily lives; those who require long-term care level 4 need complete help in their daily lives but do not have communication difficulties; and those who require long-term care level 5 need complete help in their daily lives and are bedridden [9]. The validity of these levels was previously examined in one study, and the indexed items were found to correlate well with the Barthel Index, an internationally accepted indicator for activities of daily living [11]. PD patients require care from both healthcare insurance and LTCI depending on the severity of their condition.

PD patients need to visit a physician regularly and adhere to treatment, since they are mainly treated with medication. However, the question of how physician visit frequency for PD treatment influences survival time, the number of healthcare days such as inpatient and outpatient days, and costs for all diseases, especially PD, has not been evaluated.

Hence, the aim of this study was to assess the effect of physician visit frequency for PD treatment on mortality, inpatient and outpatient days, and healthcare and long-term care costs, following the onset of PD.

## Methods

### Data

We utilized a database of healthcare claims data from the Fukuoka Prefecture Wide-Area Association of the Latter-Stage Elderly Healthcare Insurance (LSEHI) and long-term care claims data from the Fukuoka Prefecture Wide-Area Association of the LTCI, from April 2014 to March 2019. These databases contain cost information for each beneficiary.

The LSEHI is an insurer for all individuals aged ≥75 years and those between 65 and 74 years who have a certain level of disorder. Each prefecture administrates this insurance, and the Fukuoka prefecture had 613,952 beneficiaries as of March 2015 [12]. The LSEHI database includes monthly healthcare data per person, such as disease diagnosis, healthcare procedures, medication, and healthcare costs.

The LTCI includes all individuals aged ≥40 years in Japan, who can receive services once approved should they need care for any reason when they are aged 65 years or older. As mentioned, there are seven care levels, with higher levels meaning people require more help. These levels are first determined by a computer, based on the examination of the application and the report from the patient’s doctor, after which the committee for certification reviews and confirms the right level of care. The limitation of long-term care services increases as the long-term care level rises. In Fukuoka prefecture, the number of approved individuals was 39,499 as of March 2019, with 33.0% requiring help and 32.0% with care level ≥ 3 [13]. The LTCI database includes monthly long-term care data per person, such as long-term care level, care details, and total long-term care costs. The database used in this study contains anonymized individual numbers from the LSEHI; thus, we linked these two databases using these anonymized numbers.

The names, residential addresses, and individual numbers of all beneficiaries were deidentified by constructing specific databases using a secured workstation (i.e., not connected to any network and within a locked room).

### Study design and participants

This retrospective cohort study was conducted in Fukuoka, Japan. Patients included were those newly diagnosed with PD in the 2014 fiscal year (April 1, 2014–March 31, 2015). The participants were aged ≥75 years with a confirmed diagnosis using the ICD-10 code (G20: PD) and current prescription of antiparkinsonian agents using the therapeutic category code (116: antiparkinsonian agents). Those aged 65 to 74 were excluded to avoid selection bias because the insurance scheme and its coverage are conditional; only those with a specific disability are eligible to join the insurance. We gathered data from the first day of their PD diagnosis up to March 31, 2019. Data were also gathered from patients who lost their insurance eligibility, due to and up to their death.

### Statistical analysis

The distributions of sex, age category, residential facility, long-term care level, and comorbidity (malignancy, ischemic heart disease, cerebrovascular disease, dyslipidemia, diabetes mellitus, and dementia) by physician visit frequency for PD treatment were examined using chi-square tests.

Physician visit frequency for PD treatment was divided into two groups: a higher frequency of physician visits and a lower frequency of physician visits. According to a 2014 report by the Ministry of Health, Labour and Welfare of Japan, that calculated the number of days that antiparkinsonian agents were prescribed for per one physician visit, approximately 70% of patients were prescribed antiparkinsonian agents for ≤30 days, and 90% were prescribed for ≤60 days [14]. In Japan, because medication can only be obtained for the specific amount prescribed, most patients were assumed to need to visit a physician at least once every 2 months. As the guidelines for PD in Japan do not specify the number of days for which medication should be prescribed for and healthcare claims data are compiled monthly, physician visit frequency was defined as a patient’s number of months that PD treatment was claimed for divided by the number of follow-up months: a higher frequency of physician visits was defined if the number of months of PD treatment divided by the number of follow-up months was ≥0.5, and lower frequency of physician visits if < 0.5. The age categories were divided into four groups: 75–79, 80–84, 85–89, and ≥ 90 years, reflecting the onset of PD. Residential facilities were categorized into home, retirement home, long-term care facility, and healthcare institute. Patients who received inpatient care for > 28 days per month were assumed to have lived in a healthcare institute. The long-term care levels were categorized into four levels: none, requiring help that can prevent long-term care needs, long-term care levels 1–2, and long-term care levels 3–5. Comorbidity refers to an identified prevalent disease in older adults, and we extracted the records of each disease without suspicion from the onset of PD to the end of this study period. A diagnosis of dementia was understood to include Lewy body dementia.

Survival analysis was conducted to evaluate the mortality of the study participants using the Kaplan-Meier method and generalized Wilcoxon test to compare higher and lower frequency of physician visits, based on survival months. Censoring was present due to participants becoming ineligible for insurance, such as moving outside of Fukuoka. Unadjusted and adjusted restricted mean survival time (RMST) were calculated to examine the effect of physician visit frequency and variables on RMST, and RMST differences, ratios, and 95% confidence intervals were also calculated. The RMST is a summary measure of the survival time distribution *μ*, defined as the area under the curve of the survival function up to a truncation time point τ (≤60), where *S(t)* is the survival function for time *t* for integration *dt* [15]. The function is as follows:

$$\mu ={\int}_0^{\tau }S(t) dt$$

The covariates were sex, age category, residential facility, long-term care level, and comorbidities. The reference measures were lower frequency of physician visits, male, aged 75–79 years, home resident, no long-term care level, and no comorbidity.

Generalized linear models (GLMs) with gamma distribution were constructed to evaluate healthcare services, healthcare, and long-term care costs among each variable. We calculated the number of inpatient and outpatient days, costs of healthcare services, and long-term care costs per month, using records from the database during this study period and the number of follow-up months. The independent variables were physician visit frequency, sex, age category, residential facility, long-term care level, mortality, and comorbidity. The reference measures were a lower frequency of physician visits, male, aged 75–79, home resident, no long-term care level, no morbidity, and no comorbidity. Following these analyses, we calculated the marginal means of days (each separately: inpatient and outpatient days for all diseases, inpatient and outpatient days for PD) and marginal means of cost (each separately: total of healthcare and long-term care costs, healthcare and inpatient costs for all diseases, healthcare and inpatient costs for PD, and long-term care costs, USD 1 = JPY 110) per month, per participant. Inpatient and outpatient days were assumed, except for participants with healthcare institutes for resident facilities.

We used Microsoft SQL server Management Studio 18 software to extract the data and Stata BE 17.0 (StataCorp LLC, College Station, TX, USA) for the analyses.

This study was approved by the Institutional Review Board of Kyushu University (Clinical Bioethics Committee of the Graduate School of Healthcare Sciences, Kyushu University).

## Results

**Fig. 1:**
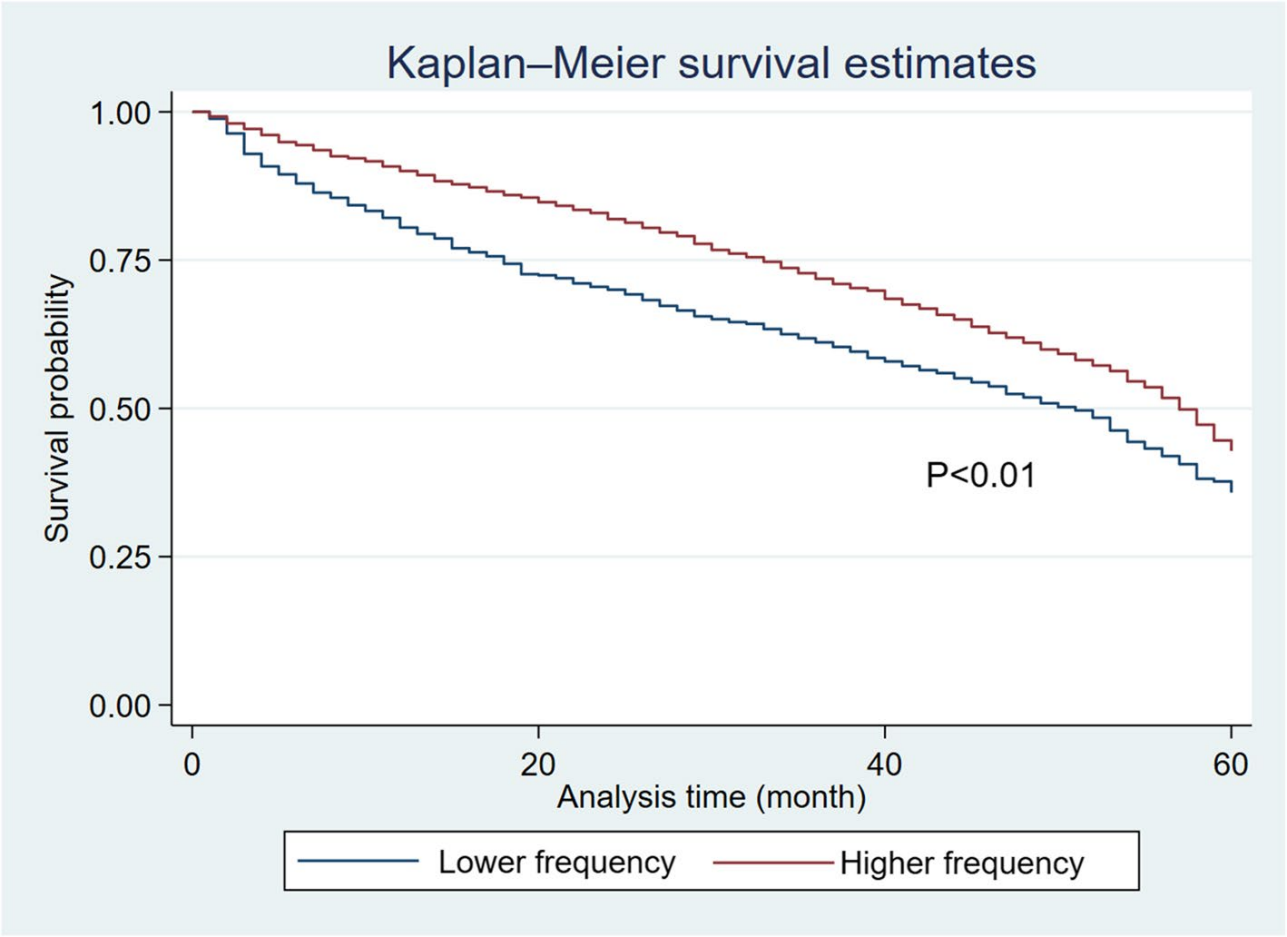
Comparison of survival time to death based on physician visit frequency for Parkinson’s disease treatment, using the Kaplan-Meier method

The total number of study participants was 2224; 1179 had a higher frequency of physician visits and 1045 had a lower frequency of physician visits. During the study period, 1137(51.6%) of participants died; of those, 548 (46.5%) had a higher frequency of physician visits, and 589 (56.4%) had a lower frequency of physician visits. The results are shown in Table 1. The results of comparing the survival time to death using the Kaplan-Meier method are shown in Fig. 1. It illustrates that patients with a higher frequency of physician visits survived longer compared to those with a lower frequency of physician visits, particularly in the 24 months after the onset of PD. After the generalized Wilcoxon test was conducted, patients with a higher frequency of physician visits had significantly longer survival times (*P* < 0.01). Table 2 shows the results of the RMST calculation. The truncation time point was defined at 24 months from the Kaplan-Meier survival curve and at 60 months, the end of the study period. The results showed that patients with a higher frequency of physician visits had a longer survival time, corresponding to 1.98 months for unadjusted and 1.57 months for adjusted covariates at 24 months, and to 5.72 months for unadjusted and 5.00 months for adjusted covariates at 60 months after the onset of PD. The ratio of RMST for physician visit frequency was 1.08 (95% confidence interval 1.05–1.11) at 24 months and 1.12 (95% confidence interval 1.06–1.19) at 60 months. Evaluating the influence of other covariates showed that being female, younger, having a lower long-term care level, having dyslipidemia, and having dementia were associated with longer survival time, while living in a residential facility and having ischemic heart disease, cerebrovascular disease, and diabetes mellitus did not show significant differences. Although malignancy was associated with a significantly shorter survival time at 24 months, there were no significant differences at 60 months.

**Table 1:** Distributions of participants’ characteristics by physician visit frequency for PD treatment using chi-square tests

	Higher frequency	Lower frequency	Total	*P*-value
Total	1045	1179	2224	
Sex				0.31
Male	430 (41.1%)	460 (39.0%)	890 (40.0%)	
Female	615 (58.9%)	719 (61.0%)	1334(60.0%)	
Age				< 0.01
75–79	291 (27.8%)	371 (31.5%)	662 (29.8%)	
80–84	348 (33.3%)	452 (38.3%)	800 (36.0%)	
85–89	284 (27.2%)	246 (20.9%)	530 (23.8%)	
≥ 90	122 (11.7%)	110 (9.3%)	232 (10.4%)	
Residential facility				< 0.01
Home	734 (70.2%)	939 (79.6%)	1673 (75.2%)	
Retirement home	31 (3.0%)	54 (4.6%)	85 (3.8%)	
Long-term care facility	184 (17.6%)	184 (15.6%)	368 (16.5%)	
Health care institute	96 (9.2%)	2 (0.2%)	98(4.4%)	
Long-term care level				0.04
None	437 (41.8%)	506 (42.9%)	943 (42.4%)	
Requiring help	96 (9.2%)	147 (12.5%)	243 (10.9%)	
Long-term care level 1–2	248 (23.7%)	265 (22.5%)	513 (23.1%)	
Long-term care level 3–5	264 (25.3%)	261 (22.1%)	525 (23.6%)	
Comorbidity				
Malignancy	259 (24.8%)	292 (24.8%)	551 (24.8%)	0.99
Ischemic heart disease	459 (43.9%)	528 (44.8%)	987 (44.4%)	0.68
Cerebrovascular disease	756 (72.3%)	808 (68.5%)	1564 (70.3%)	0.05
Dyslipidemia	473 (45.3%)	610 (51.7%)	1083 (48.7%)	0.00
Diabetes mellitus	443 (42.4%)	508 (43.1%)	951 (42.8%)	0.74
Dementia	675 (64.6%)	735 (62.3%)	1410 (63.4%)	0.27
Mortality	589 (56.4%)	548 (46.5%)	1137 (51.1%)	< 0.01

**Table 2:** RMST results

	Differences in RMST					Ratio of RMST				
	Difference	95% CI			*P*-value	Ratio	95% CI			*P-value*
24 months										
Unadjusted	1.98	1.42	–	2.55	<0.01	1.10	1.07	–	1.13	<0.01
Adjusted										
Intercept	19.99	18.41	–	21.56	<0.01	19.93	18.43	–	21.55	<0.01
Physician visit frequency for PD treatment	1.57	1.05	–	2.09	<0.01	1.08	1.05	–	1.11	<0.01
Sex	1.97	1.42	–	2.52	<0.01	1.10	1.07	–	1.13	<0.01
Age	−1.01	−1.32	–	−0.71	<0.01	0.95	0.94	–	0.97	<0.01
Residential facility	−0.29	− 0.65	–	0.07	0.11	0.99	0.97	–	1.00	0.11
Long-term care level	−1.17	− 1.43	–	− 0.91	<0.01	0.95	0.93	–	0.96	<0.01
Malignancy	−1.04	− 1.66	–	−0.42	<0.01	0.95	0.92	–	0.98	<0.01
Ischemic heart disease	0.23	−0.29	–	0.75	0.39	1.01	0.99	–	1.04	0.37
Cerebrovascular disease	0.40	−0.21	–	1.00	0.20	1.02	0.99	–	1.05	0.22
Dyslipidemia	1.37	0.85	–	1.90	<0.01	1.07	1.04	–	1.10	<0.01
Diabetes mellitus	0.45	−0.07	–	0.96	0.09	1.02	1.00	–	1.05	0.08
Dementia	1.61	1.04	–	2.17	<0.01	1.08	1.05	–	1.11	<0.01
60 months										
Unadjusted	5.72	3.98	–	7.46	<0.01	1.14	1.10	–	1.19	<0.01
Adjusted										
Intercept	45.74	37.25	–	54.22	<0.01	45.46	36.81	–	56.14	<0.01
Physician visit frequency for PD treatment	5.00	2.78	–	7.22	<0.01	1.12	1.06	–	1.19	<0.01
Sex	7.66	4.68	–	10.64	<0.01	1.20	1.11	–	1.29	<0.01
Age	−4.51	−5.95	–	−3.08	<0.01	0.90	0.87	–	0.93	<0.01
Residential facility	−1.72	−3.44	–	0.01	0.05	0.96	0.91	–	1.00	0.06
Long-term care level	−3.75	−5.12	–	−2.38	<0.01	0.92	0.88	–	0.95	<0.01
Malignancy	−3.42	−6.85	–	0.01	0.05	0.93	0.85	–	1.01	0.07
Ischemic heart disease	0.70	−2.13	–	3.53	0.63	1.01	0.95	–	1.08	0.69
Cerebrovascular disease	−0.25	−3.43	–	2.92	0.88	0.99	0.92	–	1.07	0.85
Dyslipidemia	4.66	1.71	–	7.61	<0.01	1.11	1.04	–	1.19	<0.01
Diabetes mellitus	0.09	−2.75	–	2.93	0.95	1.00	0.94	–	1.07	0.99
Dementia	4.59	1.59	–	7.58	<0.01	1.12	1.04	–	1.20	

Abbreviation: *CI* confidence interval, *RMST* restricted mean survival time

The GLMs results in Tables 3 and 4 show that a higher frequency of physician visits was associated with a significant decrease in inpatient days for all diseases, inpatient days for PD, total healthcare and long-term care costs, healthcare costs for all diseases, inpatient healthcare costs for all diseases, and inpatient healthcare costs for PD (compared with a lower frequency of physician visits). However, the number of outpatient days for all diseases and for PD was associated with a significant increase. Healthcare costs for PD and long-term care costs did not show significant differences between higher and lower frequencies of physician visits. Additionally, Tables 3 and 4 show the respective marginal means of healthcare days and healthcare and long-term care costs per month, per participant, estimated from the GLMs. Comparing higher and lower frequencies of physician visits, the inpatient days for all diseases were 3.13 days and 7.99 days, and those for PD were 1.99 days and 3.98 days. The total healthcare and long-term care costs were USD 3037 and USD 3922, respectively. The total healthcare costs for PD and long-term care costs did not significantly differ between them.

**Table 3:** The marginal means of healthcare days per month per participant estimated from GLMs

	Inpatient days for all diseases		Outpatient days for all diseases		Inpatient days for PD		Outpatient days for PD	
	Coefficient	*P-value*	Coefficient	*P-value*	Coefficient	*P-value*	Coefficient	*P-value*
GLMs^a^	−0.94	< 0.01	0.46	< 0.01	−0.69	< 0.01	1.93	< 0.01
	Days	(SE)	Days	(SE)	Days	(SE)	Days	(SE)
Physician visit frequency for PD treatment								
Lower frequency	7.99	(0.28)	2.35	(0.08)	3.98	(0.22)	0.31	(0.01)
Higher frequency	3.13	(0.11)	3.74	(0.10)	1.99	0.11	2.15	(0.06)
Sex								
Male	5.49	(0.21)	3.05	(0.12)	2.88	(0.17)	1.33	(0.05)
Female	5.46	(0.19)	3.18	(0.08)	3.02	(0.16)	1.31	(0.04)
Age								
75–79	5.93	(0.30)	3.19	(0.14)	3.10	(0.25)	1.30	(0.06)
80–84	5.45	(0.23)	3.18	(0.10)	2.96	(0.19)	1.37	(0.06)
85–89	5.79	(0.27)	2.81	(0.11)	3.27	(0.24)	1.26	(0.06)
≥ 90	4.23	(0.30)	3.45	(0.27)	2.18	(0.25)	1.36	(0.11)
Residential facility								
Home	5.94	(0.18)	3.13	(0.08)	3.25	(0.16)	1.30	(0.04)
Retirement home	5.65	(0.63)	3.34	(0.39)	2.85	(0.52)	1.35	(0.14)
Long-term care facility	4.09	(0.24)	3.05	(0.18)	2.15	(0.19)	1.38	(0.09)
Long-term care level								
None	4.96	(0.23)	3.26	(0.13)	2.62	(0.18)	1.23	(0.05)
Requiring help	6.36	(0.46)	3.10	(0.15)	3.81	(0.40)	1.22	(0.07)
Long-term care level 1–2	5.44	(0.28)	2.86	(0.11)	3.09	(0.24)	1.26	(0.06)
Long-term care level 3–5	5.83	(0.29)	3.16	(0.20)	2.96	(0.23)	1.60	(0.10)
Mortality								
No	3.90	(0.21)	3.29	(0.12)	2.25	(0.18)	1.27	(0.05)
Yes	6.30	(0.20)	2.95	(0.10)	3.30	(0.16)	1.37	(0.06)
Malignancy								
No	5.36	(0.16)	3.00	(0.08)	3.05	(0.14)	1.37	(0.04)
Yes	5.76	(0.28)	3.52	(0.14)	2.74	(0.20)	1.19	(0.06)
Ischemic heart disease								
No	5.15	(0.18)	2.90	(0.09)	2.74	(0.14)	1.24	(0.04)
Yes	5.89	(0.22)	3.40	(0.10)	3.25	(0.19)	1.42	(0.06)
Cerebrovascular disease								
No	4.62	(0.23)	3.12	(0.15)	2.81	(0.19)	1.28	(0.05)
Yes	5.83	(0.17)	3.13	(0.07)	3.02	(0.14)	1.34	(0.04)
Dyslipidemia								
No	5.82	(0.19)	2.93	(0.09)	3.24	(0.16)	1.38	(0.05)
Yes	5.02	(0.20)	3.31	(0.11)	2.58	(0.16)	1.26	(0.04)
Diabetes mellitus								
No	5.49	(0.18)	2.90	(0.08)	3.07	(0.15)	1.27	(0.04)
Yes	5.45	(0.21)	3.41	(0.12)	2.80	(0.17)	1.39	(0.06)
Dementia								
No	4.55	(0.20)	3.00	(0.09)	2.31	(0.15)	1.19	(0.05)
Yes	6.01	(0.19)	3.21	(0.10)	3.35	(0.16)	1.40	(0.05)

^a^GLMs only shows the result of physician visit frequency for PD treatment

Abbreviation: *SE* Standard error

**Table 4:** The marginal means of healthcare and long-term care costs per month per participant estimated from GLMs

	Total of healthcare and long-term care costs		Healthcare costs for all diseases		Inpatient healthcare costs for all diseases		Healthcare costs for PD		Inpatient healthcare costs for PD		Long-term care costs	
	Coefficient	*P-value*	Coefficient	*P-value*	Coefficient	*P-value*	Coefficient	*P-value*	Coefficient	*P-value*	Coefficient	*P-value*
GLMs^a^	−0.26	< 0.01	−0.40	< 0.01	−0.70	< 0.01	−0.01	0.90	−0.52	< 0.01	0.01	0.91
	Costs	(SE)	Costs	(SE)	Costs	(SE)	Costs	(SE)	Costs	(SE)	Costs	(SE)
Physician visit frequency for PD treatment												
Lower frequency	3922	(84)	2382	(50)	2153	(59)	1041	(43)	1029	(47)	1483	(83)
Higher frequency	3037	(62)	1594	(37)	1065	(39)	1033	(46)	612	(39)	1496	(61)
Sex												
Male	3414	(76)	2015	(46)	1689	(55)	1002	(43)	832	(47)	1436	(86)
Female	3523	(71)	2011	(44)	1652	(53)	1068	(40)	876	(46)	1518	(61)
Age												
75–79	3469	(112)	2176	(67)	1828	(82)	1062	(57)	880	(64)	1321	(111)
80–84	3604	(87)	2056	(52)	1696	(65)	1067	(48)	883	(55)	1541	(84)
85–89	3557	(100)	2001	(58)	1710	(70)	1100	(57)	930	(62)	1569	(101)
≥ 90	3040	(110)	1638	(74)	1279	(82)	812	(65)	616	(66)	1476	(135)
Residential facility												
Home	3268	(57)	1958	(38)	1559	(42)	954	(32)	757	(33)	1330	(54)
Retirement home	3771	(266)	1953	(145)	1548	(162)	940	(118)	683	(116)	1738	(225)
Long-term care facility	3729	(123)	1431	(55)	1087	(58)	722	(46)	517	(44)	2131	(117)
Health care institute	4959	(211)	4827	(265)	4855	(357)	3510	(395)	3129	(394)	13	(5)
Long-term care level												
None	2655	(74)	1903	(49)	1545	(59)	966	(46)	788	(50)	723	(54)
Requiring help	3836	(199)	2275	(109)	2014	(144)	1263	(103)	1163	(124)	1792	(220)
Long-term care level 1–2	4014	(106)	2004	(64)	1678	(77)	1068	(60)	905	(68)	1886	(99)
Long-term care level 3–5	3966	(101)	2093	(68)	1733	(80)	1038	(57)	822	(62)	1936	(110)
Mortality												
No	2908	(94)	1656	(52)	1180	(54)	881	(50)	651	(50)	1314	(90)
Yes	3852	(72)	2210	(44)	1902	(51)	1112	(39)	939	(43)	1647	(91)
Malignancy												
No	3479	(60)	1952	(37)	1615	(43)	1058	(36)	872	(40)	1550	(54)
Yes	3473	(95)	2181	(60)	1817	(74)	980	(49)	807	(55)	1287	(98)
Ischemic heart disease												
No	3312	(63)	1897	(40)	1555	(47)	991	(37)	800	(39)	1447	(63)
Yes	3695	(86)	2168	(48)	1829	(61)	1104	(46)	936	(54)	1546	(83)
Cerebrovascular disease												
No	3281	(99)	1786	(52)	1419	(59)	1022	(48)	829	(52)	1497	(103)
Yes	3559	(58)	2111	(39)	1778	(49)	1045	(37)	867	(41)	1487	(52)
Dyslipidemia												
No	3580	(68)	2055	(42)	1724	(50)	1108	(40)	914	(44)	1500	(71)
Yes	3349	(80)	1957	(47)	1589	(57)	938	(41)	763	(45)	1479	(82)
Diabetes mellitus												
No	3438	(67)	1943	(39)	1629	(47)	1042	(36)	866	(40)	1499	(67)
Yes	3535	(79)	2119	(50)	1734	(61)	1031	(46)	834	(51)	1478	(71)
Dementia												
No	3034	(78)	1908	(51)	1495	(58)	928	(43)	713	(44)	993	(59)
Yes	3704	(69)	2075	(39)	1773	(50)	1103	(38)	941	(45)	1698	(63)

Costs were converted from Japanese yen to US dollars (USD 1 = JPY 110)

Abbreviation: *SE* Standard error

^a^GLMs only shows the result of physician visit frequency for PD treatment

## Discussion

This study focused on the physician visit frequency for PD treatment for people newly diagnosed with PD among older adults in Japan, and evaluated mortality, healthcare days, and costs for healthcare and long-term care. We found that a higher frequency of physician visits was significantly associated with longer survival time, decreased inpatient days, and reduced healthcare costs, considering other variables. In other words, high physician visit frequency for treatment, including prescribed medicine or adjusted treatment, contributed to prolonging PD patients’ lives. These results suggest that PD patients would benefit from the support of caregivers, including healthcare professionals and family members, to visit a physician regularly. However, the findings and suggestions based on the findings may not be generalizable to other health care systems outside Japan wherein the prescription of drugs does not require physician visits. A previous study showed that the median length of stay in healthcare institutes among PD patients has steadily declined over the last 10 years [16] but is still longer than that of the general population [17]. Other studies have concluded that PD patients have higher rates of emergency admissions with longer hospital stays, higher costs, and more in-hospital mortality than all other admissions [18]. Japan established a community-based integrated care system in which older adults can live the rest of their lives according to their preferences in environments familiar to them, even if they were to become heavily in need of long-term care. Regardless of what disease patients were facing, those who were receiving care from enhanced home care support healthcare institutes had relatively few hospitalizations, low in-hospital mortality, and a high utilization of home care and home-based end-of-life care [19]. The same is true of PD patients who could continue to live where they preferred when they utilized home care support from these healthcare institutes.

Other factors with a negative influence on survival time were sex (male), older age at the time of PD diagnosis, and higher levels of long-term care. Previous studies of PD also revealed that being male [4, 20], older [4, 20, 21], and experiencing severe motor symptoms [21] and frailty [22] were associated with mortality. Although the present study only included newly diagnosed patients, a higher long-term care level at the time of diagnosis showed a shorter survival time even if the estimates were adjusted for age and comorbidity. Drug treatment in patients with early-stage PD has been found to increase health state values [23]. In the current study, malignancy as a comorbidity was associated with decreased survival time, whereas the presence of dyslipidemia was related to increased survival time. Regular usage of statins, a drug for dyslipidemia, may reduce the risk of PD, but no difference in efficacy has been observed in older patients [24]. Nonetheless, the risk of PD among patients with diabetes using statins is lower than that among nonusers [25]. However, we did not evaluate the effect of statins in this study. Having dementia was associated with a longer survival time, and other comorbidities were not associated with mortality in the current study. Nonetheless, a previous study suggested an association between dementia, cardiovascular diseases, and cerebrovascular diseases among PD patients and increased mortality [5, 20]. As this study did not evaluate the time of diagnosis and severity of each comorbidity, these factors might have influenced the results. Moreover, the results obtained for participants with dementia were inconsistent with those of previous studies; however, this could be due to social factors, such as life events and social networks. Further, administering exenatide, a drug for diabetes mellitus, has been shown to have a positive effect on PD patients [26], but no participants in this study were prescribed this drug during the study period. This study showed that a higher long-term care level at the time of PD diagnosis was associated with higher mortality. The current study’s participants were older adults and 10.4% of them were diagnosed when they were already over 90 years old. Therefore, these participants might have already had advanced-stage PD before diagnosis because symptoms at the early stage of PD are similar to those of normal aging. Consequently, based on the current study’s findings, it is important that patients are diagnosed at an early stage, visit a physician regularly, and follow appropriate treatment.

Healthcare costs for PD can be partially covered by the government in Japan when the severity level becomes ≥3 according to the Hoehn and Yahr stage, and the level of functioning and disability becomes ≥2 (requiring partial assistance in daily life and physician visits). The number of patients covered by this system increases every year, being 127,536 in March 2018 [27]. Approximately 21.3% of PD patients (27.3% for patients aged ≥75 years) do not use this system. Thus, those PD patients, particularly older adults, are assumed to have mild PD. According to research in the United States, the length of hospital stay for PD patients has been declining over the last 10 years; however, as the cost of care has been rising [16], the economic burden of PD is increasing [28, 29]. The cost of PD per patient varies between countries [28, 30]. The current study did not evaluate the economic burden but estimated costs per month per PD patient. Nonetheless, we can assume that an increasing number of PD patients would coincide with an increasing economic burden in Japan. As this study showed that monthly healthcare costs per PD patient were lower among those with a higher frequency of physician visits than those with a lower frequency of physician visits, it is important for caregivers, including healthcare professionals and family members, to support PD patients to attend regular physician visits to help decrease the economic burden of this disease.

Regarding other variables, residential facilities were not associated with survival time but were associated with inpatient days and healthcare and long-term care costs. PD patients living at home tended to have longer hospitalizations and higher inpatient costs compared to patients in retirement homes and long-term care facilities, while total costs were lower considering long-term care costs. Higher long-term care levels have higher long-term care costs, but there were no significant differences in healthcare costs among long-term care levels considering age. The long-term care levels of participants with no long-term care level at the time of PD diagnosis might have increased during the study period, but the number of inpatient days and healthcare costs per month were still lower than those for participants with other long-term care levels. Thus, they might have maintained lower long-term care levels because their long-term care costs also stayed lower.

This study has some limitations. First, physician visit frequency should not be interpreted as patient adherence as this construal might be subject to misclassification. Some PD patients might be classed as having a lower frequency of physician visits despite adhering to the treatments; this is because they might have been prescribed medicine that lasts for more than 60 days per physician visit. Conversely, other PD patients might be classed as having a higher frequency of physician visits even though their physicians prescribed them medicine that lasts less than 30 days and they did not visit their physicians until after that. As this study has utilized medical claims data, we could not evaluate actual individual adherence. Second, other factors associated with mortality could not be evaluated, such as healthy life expectancy and nutritional status, because this study utilized claims data. Nutritional status is an important factor for health among older people and is related to body mass index. While body mass index is not associated with mortality in PD patients in the United States [21], it may be associated in Japan [31]; however, we could not clarify this association because of data limitations, so future studies should address this aspect. In addition, we could not evaluate the onset and severity of comorbidities. Older patients with dementia are known to have low levels of medication adherence [32]. Taking this information into account could lead to different results regarding the influence of comorbidities on mortality. Third, we did not have information on whether study participants lived at home alone or with other family members, which is likely to affect physician visit frequency for treatment. Lastly, we could not evaluate the utilization of home-visit nurse services because of claim systems differences in Japan: home-visit nurse services offered by healthcare institutes and home-visit nurse institutes differ, as do the methods for claims. In addition, home-visit nurse services can normally be obtained through either healthcare insurance or LTCI, but PD patients must utilize healthcare insurance because of their dependence on treatment from healthcare. However, no current electronic data for such services provided by home-visit nurse institutes utilizing healthcare insurance yet exist. Many countries are more interested in the activities of nurses in promoting home-based care systems. For example, in the Netherlands, a guideline for PD nurse specialists is provided [33], and research on specialized nursing interventions for PD patients is being planned [34]. The Japanese government is planning to create electronic data for home-visit nurse services from home-visit nurse institutes utilizing healthcare insurance in 2024. In addition, Japan has established a community-based integrated care system to offer comprehensive care, including home-based healthcare and long-term care, so further research is required that considers such care, including home-visit nurse services.

## Conclusions

A higher frequency of physician visits for PD treatment was significantly associated with longer survival time, fewer inpatient days, and reduced healthcare costs for PD patients. This study suggests that a system supporting patients who need continuous drug treatment, such as those with PD, to ensure physician visits for treatment through support from their caregivers, including healthcare professionals and family members, may be required. Patients with PD may live for longer if such a system is implemented.

## Data Availability

The data that support the findings of this study are available from the Fukuoka Prefecture Wide-Area Association of the Latter-Stage Elderly Healthcare Insurance and the Fukuoka Prefecture Wide-Area Association of the Long-term Care Insurance, but restrictions apply to the availability of these data, which were used under license for the current study and so are not publicly available. The data are, however, available from the authors upon reasonable request and with permission from these insurance companies.

## Abbreviations

PD: Parkinson’s disease
LTCI: Long-Term Care Insurance
LSEHI: Latter-Stage Elderly Healthcare Insurance
GLMs: Generalized linear models
